# Supplementary material for: Dimensionality, information and learning in prefrontal cortex
Source: PLoS Comput Biol. 2020 Apr 24;16(4):e1007514. doi: 10.1371/journal.pcbi.1007514 (PMC7202668; doi:10.1371/journal.pcbi.1007514)
Supplement: Attachment — Submitted filename: Reply to reviewers PLOS CB 02.docx [file pcbi.1007514.s001.docx]

We would like to thank the reviewers for their support of our manuscript, and for their thorough, detailed and insightful comments. We have addressed each of the comments and feel that they have led to important clarifications and additional analyses which provide additional insight into the data.

Reviewer 1

Comment 1. Page 7: clarify: add neuron index to U=[mu…] – to emphasize you have many such vectors. Also (in the same sentence) does mean activity mean: trail average of spike count?
The matrix U is 192 times N, where N is the number of neurons. You should better motivate doing SVM on the 192 dimensions and not on the N dimensions, and explain its meaning (you are doing SVM on vectors that the ‘tuning curves’ of different neurons).

Reply: We have added a phrase to indicate that this is trial averaged spike count. Each mu is a vector and each element of the vector is a neuron, which we had not made clear. We have now clarified this. “The mean activity (i.e. trial averaged spike count) was further estimated in two, 250 ms bins, time locked to cue onset (Fig. 3A; *x_1_* = 1-250 ms and *x_2_* = 251-500 ms) for each neuron. We collected these mean estimates for all neurons into vectors, $\mu,$ and collected the vectors into a matrix, $U=[\mu_{1,1,1,1}\ldots\mu_{l,m,n,o}\ldots\mu_{2,2,2,24}]$. The matrix *U* had 192 columns because each column of this matrix was one mean response vector $\mu$ (where the indices take on values *l =* 2 time bins x *m* = 2 choices x *n =* 2 phases x *o =* 24 blocks; see methods). The number of rows in *U* was given by the number of neurons simultaneously recorded. The maximum dimensionality in our data was, therefore, 192, because of the number of conditions analyzed.”

The eigenspectrum of our data is the same whether we extract it from the Neuron x Neuron covariance matrix or the Condition by Condition covariance matrix, and the maximum number of non-zero eigenvalues is 192. We have now added a comment to this effect, with a reference, This dimensionality is the same if one extracts eigenvectors from the Neuron x Neuron covariance matrix or the Conditions x Conditions covariance matrix [6].

The actual eigenvectors extracted differ depending upon which matrix we use. In fact, SVD factors the matrix U, but one of the matrices of singular vectors is equivalent to the eigenvectors extracted from the covariance matrix. We do not use the eigenvectors, and only examine the eigenspectrum.

Comment 2. Figure 3D: the matrix W has only 96 columns. How can you show 100 eigen dimensions?

Reply: This is slightly confusing. The matrix has 96 independent dimensions, so the cumulate variance saturates at 1, in the 96^th^ dimension. We have added a note to the legend in Figure 3 to clarify this. For the analysis we extract eigen dimensions from the Neuron x Neuron covariance matrix. So the analysis returns N_neurons dimensions, but only the first 96 are independent.

Comment 3. Fig 3C-H consider comparing with shuffled data (along the neuron dimension) of the same length.

Reply: This was a good suggestion and we worked on it but could not think of a good way to do this. For the analysis we accumulate the covariance matrix by calculating outer-products of the mean centered vectors from each block *j*, i.e. sum_j w_j*w_j’. If we shuffle the neuron index of both *w*’s we get an equivalent covariance matrix. If we shuffle the neuron index of one of the *w’s*, we do not get a symmetric, positive definite matrix.

Comment 4. Fig 3F: here the ‘principle component’ contributes about 40% for where and 20% for What, and the rest seem to add much less. It seems possible that the main difference between Where/What and Random in Fig 3F may result from this contribution of the first eigen dimension.

Reply: This was a very interesting suggestion and we had not thought of this. In fact most of the difference was driven by dimension 1, which likely reflect population coding of left/right choices in the Where condition. When we removed the first dimension before calculating cumulative variance, the difference between conditions was not significant. We have added text to the results and a new panel Fig. 3I. ”Most of the difference between conditions, however, was driven by the first principal component. When this was removed in both conditions, there was no difference between conditions (Fig. 3I; F(1, 5) = 4.33, p = 0.092).”

Comment 5. Fig S1 could be incorporated into the main body of the paper, per author’s judgement (same for S2 and S3).

Reply: This was a good suggestion and we have now incorporated these figures into the main text.

Comment 6. Fig 4A – need to better explain (for example: what are the axes?)

Reply: We have decided to remove this figure panel so we can show the measured and predicted decoding performance split out by condition more clearly and keep a well-balanced figure.

Comment 7. Figure 4 C verses F – is it a ceiling effect?

Reply: This is a good question and it is possible that it is a ceiling effect. Although the information values are all quite high and an information of 25 (the x-phase value) gives a classification rate of 0.99. The difference between the pattern for information and classification could represent something real about the neural representations in reversal, but it is not clear, so we have commented on it minimally.

Comment 8. Figure 4D: could you plot the what and where using different signs using the same color code of B,C,E,F?

Reply: This was a good suggestion. Also the previous plot only showed the data for the within condition so we have added the other conditions. We tried overlaying all points for both block types but it was difficult to make out the details. So we have now included 2 figure panels, one for each condition as suggested.

Comment 9. Fig 4: did you look how information space/representation changes over to the second next block?

Reply: We have looked at this in a few different ways, although we did not exactly plot it with respect to the next block. On average, the change to the next block would be captured by x-block, if the next block is of the same condition, and x-cond if the next block is of the opposite condition. These quantities were both average over all other blocks, and not just the next block. Also, Fig. 5 (now Fig. 7) shows how delta information (closely related) drops when we transition to a new block.

Comment 10. Page 11: please clarify what you mean by “different blocks of the What condition were more independent”?

Reply: We have added the following phrase to further clarify this, “because information dropped more between the Within subspace and the other conditions for What blocks than Where blocks…”

Comment 11. Figure 5: what exactly are showing here? What is the ellipsoid? What are Dimenstion X? Why only four red points in Fig 5B? Also in Fig 5D – it seems counter intuitive that the DInformation for the X-block is around zero (orange traces?), whereas in Fig 4C&F information measures are very high.

Reply: Apologies for not clearly labeling our plots. We have added the following to the legend to clarify this. (Now Fig. 7) “Additional linked points are subsequent trials of the same choice, which are not necessarily consecutive trials in the task. Because the number of times each option was chosen in each block varies, the number of points varies. Option 1 was chosen more often than option 2 in this example block. Ellipses show 1 standard deviation of data for each condition. Dimensions 1 and 2 on the x and y axes refer to the subspace for each block, which is defined by rotations within the subspace spanned by the activity in the two time bins (i.e. 0-250 and 251-500 ms after cue onset).“

With respect to the question of the low information for x-block in the Where condition, your question is very observant. The answer is buried a bit in the methods in the definition of the quantities. When we calculated information values in Fig. 4, we re-estimated the means for each choice after projection of single trial activity into the subspace. When we calculated the dInformation values in Fig. 5, we did not re-estimate means after projection, we used the means for the other block. Thus, the activity varies along similar dimensions in other blocks of the Where condition, but the means are affected. This is also true for the reversal data. We used the means for the reversal choices. We have added a note to the legend of Fig. 5 (now Fig. 7) which now states, “Note, values for x-block ΔInformation are low because we did not re-estimate means after projection for this analysis, as we did for the analyses in Fig. 4.“

Comment 12. Fig 6E&F: from Fig 4 one expects more information in where than in what – it seems that in Fig 6 this is opposite. Can you comment?

Reply: Yes, in general this would be true. The difference comes from the use of a different covariance matrix for these analyses. We were concerned about over-fitting, so we used a few variants while exploring these data. Results were generally consistent independent of how we estimated noise. For Fig. 4 the noise was calculated using only the trials within each block and phase after projecting the data into the subspace, and doing cross validation. For Fig. 6 we used a noise covariance matrix calculated by pooling across all blocks except the current block under analysis. Thus, we had many more trials available for the covariance estimation, which led to results being more stable. When we did the analysis in Fig. 4 using the x-block cross validated covariance matrix as we did in Fig. 6, the estimated and predicted performance (i.e. the new Fig. 4A and 4D) did not match as well. Results were more stable when we used the pooled covariance matrix for the distance calculations. In Fig. 4 we did more averaging because we averaged across all trials, whereas for Fig. 6 we were splitting out by trial. So having more variability in noise estimates mattered less for Fig. 4 than Fig. 6.

Comment 13. Results of Fig 6A-D are fascinating. If you will flip dimension 1 in 6C (multiply it by -1, I think it is perfectly fair) you will get almost the same plot as in 6A (also similar to some extent in 6D and 6B) – outlines a clear trend.

Reply: Thank you. There are some additional interesting trends in the data that we did not pursue. For example, it seems that when activity re-organizes it is often for only one of the targets and not the other. For example the orange and red dots in panel c do not re-organize, but the blue dots do.

Comment 14. Page 18 typo {Oby, 2019 #6939}.

Reply: Fixed. Thanks.

Reviewer 2

General comments: The paper is overall clearly presented but would benefit from some descriptive section headings in the results.

Reply: Thank you. We have added section headings to clarify the results.

Comment 1. There is no mention of cross-validation for the computation of dimensionality (Fig 3). One way to cross-validate is to separate the trials into 3 sets, compute the eigenvectors on the covariance between set1 and set2 and then project the eigenvectors onto set3 to get the variance of the eigenvectors in an unbiased way. Can you also justify why only 2 time bins were used?

Reply: This was a good suggestion, although it is difficult to carry this out in the limited trial regime in which we operate. That was part of the motivation for examining the geometry of the population code using dimensionality, information and decoding analyses. With respect to dimensionality, because we need trials of both conditions to estimate the dimensions, and we remove the first 4 trials of each block, and monkeys learn to pick one of the options more often in each block, we often end up with blocks in which we have only a few trials of one of the choices. We discussed this in the methods when describing cross validation for the decoding and information calculations. We removed blocks in which there were not enough trials of each choice. This is also why we have focused all analyses on comparisons between What and Where, because these factors are all balanced across conditions. Our claims are specifically with respect to differences between these conditions.

However, we have carried out the requested analysis and have included a new panel (Fig. 3J) which shows the cross-validated variance accounted for in both conditions. To carry out this analysis we left out one trial from each condition. We then computed the mean across the remaining trials for each condition, and then computed *w_estimate* from these means. We then accumulated these and computed SVD, as we had done previously. We then projected the held-out trial (actually the difference between the two held-out trials, *w_test*) onto the principal components, and calculated the variance accounted for in the held-out trials. Because much of the variance in *w­_test* is going to be noise, because we are not averaging over multiple trials, *w­_test* tends to have a strong projection onto the higher eigen dimensions, and not the first few eigen dimensions. Thus, the dimensionality of the held-out trials is higher, due to the presence of noise, than the dimensionality of the covariance matrix calculated from the limited data. The difference between What and Where is still significant in the cross validated analysis. We added the following text to the results, “We also examined the variance accounted for in cross validated data. We removed one trial of each choice (e.g. one trial of choosing object 1 and one trial of choosing object 2), estimated the SVD using the remaining trials, and then projected the held-out trial onto the eigen dimensions (Fig. 3J). Note that the eigen-dimensions in these analyses (Fig. 3) were computed on the mean activity vectors from each block and phase, and when we cross-validate we are computing the variance accounted for in a single trial, as opposed to the mean across trials. Therefore, much of the variance of the single trial will be due to noise. Thus, the cross-validation analysis only captured a small amount of the variance in the held-out data. However, the What condition was still higher dimensional than the Where condition (F(1, 5) = 15.2, p = 0.012).”

We used two time bins because the relation between estimated and predicted decoding performance (Fig. 4A, D) was consistent with 2, but started to break down when we tried to use more dimensions. This was likely due to over-fitting.

Comment 2. Fig 3G,H make the claim that dimensionality increases over blocks, but there is no explanation of the null for this (dimensionality will increase as timepoints increase even for noise). I think with dimensionality computed in a cross-validated way this will be a less problematic analysis, but with un-cross-validated eigenvalues this analysis doesn’t tell us much.

Reply: We agree that the growth with blocks by itself is not that meaningful. We wanted to emphasize the difference between What and Where blocks. The dimensionality grows faster in What than Where. Since other factors, including number of trials, etc. are constant across the two conditions, the comparison is meaningful, even though the individual values are not. We have also added analysis of the reward outcomes (new Fig. 8), which further supports our claims about differences in dimensionality being driven by learning images vs. learning objects and specifically at the time of choice as opposed to throughout the trial.

Comment 3. Fig 4 again I am unsure what the control is for the F-tests. It would be nice for the reader to have a line on Fig 4B,C showing the chance amount of information for a randomly drawn vector from the subspace of responses.

Reply: We were interested in the difference in information across the subspaces defined in several ways, so the comparison is among the different definitions rather than relative to a null direction.

However, we have carried out an analysis by computing information and decoding using random directions within the space of responses as suggested (See Fig. R1). To generate random directions we weighted the response vectors from all the blocks with random draws from a standard normal Gaussian (i.e. mean 0, std = 1), and then summed the weighted vectors. We then used the resultant vector as a subspace. We plot this below as column 5, labeled “rand”. The What condition is on the left and the Where condition is on the right. Note that columns 1-4 are equivalent to columns 1-4 in Fig. 4 in the paper. The x-cond (i.e. cross condition) is a good reference, since this is the average across all of the blocks from the other condition. So they are from the response space but they are specifically from blocks of the opposite task condition.

The rand performance in the Where condition maintains a lot of information when we randomly sample directions across blocks, because Where coding is well-preserved across blocks. When we use the What blocks to decode direction, however, (i.e. x-cond), we lose considerable information, because the What directions are defined with respect to chosen objects and not chosen directions. Random directions are less useful in the What condition, however, because coding directions change more across blocks in the What condition. As we see below.

Fig. R1 – Information and decoding including random directions in response space.

Comment 4. Fig S2 needs more explanation in the figure/methods. By “each subspace” do you mean the single discriminant direction?

Reply: Per the suggestion of reviewer 1, we have moved S2 to the main text (Fig. 5A-D). We have also clarified this by adding the following to the legend: ” The subspace for a single block is given by activity in the two time bins (i.e. 0-250 ms after cue onset and 251-500 ms after cue onset).”

Comment 5. The paragraph after Fig 4 starts compellingly asking if responses depend on value, but this question is not answered until Fig 6. I am not sure what order these two figures should be in to best support the narrative, but personally I would have preferred Fig 6 before Fig 5.

Reply: Thank you for this suggestion. We did not have a strong preference for order between figures 5 and 6 (now figures 6 and 7 because we added a new figure 5). So we have switched their order as suggested.

Comment 6. Labels on Fig 5A,B axes as to what the dimensions are would be helpful to the reader. Why is Fig 5C labelled with change in information if it is a change in Mahalanobis distance? I think again it would be easier to understand after Fig 6.

Reply: We have now added text to the legend that defines the “dimension” axis labels, “Additional points are subsequent trials linked within a condition. Because the number of times each option was chosen in each block varies, the number of points varies. Option 1 was chosen more often than option 2 in this example block. Ellipses show 1 standard deviation of data for each condition. Dimensions 1 and 2 on the x and y axes refer to the subspace for each block, which is defined by rotations within the subspace spanned by mean activity in the two time bins (i.e. 0-250 and 251-500 ms after cue onset).”

The Mahalanobis distance (MD) is not often called an information measure, although the Mahalanobis distance between two distributions is equivalent to the multivariate d prime, which we use to measure information. Since we are computing the MD from a point to each of the distributions and taking the difference, we thought it accurate to call it a difference in information. We do not mind switching it to difference in distance, however, if the reviewer prefers.

Minor comments

Comment 1. You are randomly presenting the reward, but the reason for this experimental design choice is not explained. Also, this reward period activity is not evaluated at all. Is this activity low-dimensional and independent of the task condition/phase? Does it code left/right or abstract quantities?

Reply: This was an interesting suggestion. With respect to the task design, we used random reward outcomes to make learning more difficult. If we use deterministic rewards the animals get quite good and learn in 1-2 trials which option to choose. We also then get very few choices of both options in each block, and then we do not have enough trials to estimate our decoding models. In much of the work we are doing with this dataset (and in other experiments with this task) we are examining effects on learning.

As suggested we also ran analyses looking at the dimensionality of the reward signal and found that it did not differ across block types. We have added a new Fig. 8 showing this result, as we think it nicely compliments the results on the choice related activity. We also included information and decoding analyses in this figure.

Comment 2. In the discussion there is mention that coding in new subspaces prevents interference with old subspaces and object-value associations. It would be interesting to see if the subspaces in Fig 6AC project differently onto reward-related directions (Fig 6A).

Reply: Another interesting suggestion. Although we were not sure if we exactly followed the specifics of the comment. However, we projected the choice related activity onto the reward dimensions as we thought this was what was requested? (Fig R2). Information was relatively low, although decoding was still possible at a reasonable level. Note from Fig. 2 panels G and H, the information about chosen object and chosen direction are still high when the reward information starts to come in. Given that the animals learn the “best” option, choice of one of the options in each phase and block will be more often rewarded. Therefore, object/action and reward are always correlated. So we expect some information to be extractable.

Fig. R2 – Projection of choice related activity onto all dimensions shown in R1 and also the dimensions defined by reward vs. no reward outcomes (magenta bars).

Comment 3. There is a mention of reversals in which the monkey quickly learned the reversal - is this due to a chance exploration of the other choice? Is there a difference in neural activity in these periods?

Reply: This is an interesting suggestion and we are developing another manuscript to examine exactly this issue. The animals develop a sophisticated model of the task, and they tend to reverse rapidly. There are interesting features in the neural activity exactly in this reversal period. And this is what we are developing in detail. However, it is beyond the scope of the current manuscript.
 
